# Field resistance to *Orobanche crenata* in pea (*Pisum sativum* L.): beyond strigolactones

**DOI:** 10.1186/s12870-025-07296-x

**Published:** 2025-10-08

**Authors:** Francesco Arcieri, Gaetano Giudice, Marzia Guerriero, Chiara Delvento, Mario Schilder, Angelica Giancaspro, Pasquale Luca Curci, Catherine Rameau, Luigi Ricciardi, Concetta Lotti, Harro Bouwmeester, Imran Haider, Stefano Pavan

**Affiliations:** 1 Department of Soil, Plant and Food Sciences, University of Bari Aldo Moro, Bari, Italy; 2 Plant Hormone Biology Group, Swammerdam Institute for Life Sciences, University of Amsterdam, Amsterdam, Netherlands; 3 Department of Agricultural, Food and Environmental Sciences, University of Foggia, Foggia, Italy; 4 Institute of Biosciences and Bioresources, National Research Council (CNR), Bari, Italy; 5 Institut Jean-Pierre Bourgin, INRAE, AgroParisTechUniversite Paris-Saclay, Versailles, France

**Keywords:** Orobanchaceae, Pea, *Orobanche crenata*, Resistance, Strigolactones

## Abstract

**Background:**

Orobanchaceae are parasitic weeds causing substantial yield losses in many crops, including pea (*Pisum sativum* L.). Within host species, genotypes that display enhanced resistance to Orobanchaceae often exude low levels of strigolactones (SLs) from their roots, in line with evidence that SLs stimulate the germination of Orobanchaceae. However, to which extent genetically determined low SL exudation contributes to field resistance to Orobanchaceae was poorly investigated. Here, we studied the relation between SL exudation and field response to *Orobanche crenata* Forsk. (*Oc*) in pea.

**Results:**

The screening of a germplasm panel identified three novel sources of field resistance to *Oc* and revealed an association between field resistance and low SL exudation. Although the SL-deficient mutants *rms1* and *rms5* were more resistant than their wild-type backgrounds, they still suffered substantial *Oc* parasitization. Genetic analysis and RNA-seq of recombinant inbred lines uncovered both SL-dependent and SL-independent mechanisms contributing to the near-complete resistance to *Oc* previously reported in the breeding line ROR12, and identified candidate genes possibly underlying resistance loci.

**Conclusions:**

Besides identifying novel sources of resistance to *Oc*, our study indicates that reduced or absent SL exudation alone is not sufficient to confer complete field resistance to *Oc* in pea. This suggests the necessity of exploring SL-independent resistance mechanisms for breeding purposes. Further investigations are needed to clarify whether a similar scenario applies to other crops affected by Orobanchaceae, and to characterize genes causally related to *Oc* resistance.

**Supplementary Information:**

The online version contains supplementary material available at 10.1186/s12870-025-07296-x.

## Background

Orobanchaceae include root parasitic weeds, witchweeds (*Striga* spp.) and broomrapes (*Orobanche* and *Phelipanche* spp.), causing agricultural losses of about 10 billion US dollars annually [1]. Crenate broomrape (*Orobanche crenata* Forsk.) (*Oc*) is a major threat to the cultivation of cool-season legumes in the Mediterranean area, and may cause up to complete yield loss in pea (*Pisum sativum* L.), one of the most important legume species globally with a production of about 35 million metric tons [2–5].

Orobanchaceae are extremely difficult to control using agronomic and chemical methods, as their life cycle mostly occurs underground and they produce large amounts of small, long-lived seeds [6]. No commercially available pea cultivar displays significant resistance to *Oc*, therefore confining pea cultivation to non-infested areas [7–9].

Strigolactones (SLs) are carotenoid-derived compounds exuded by the roots of land plants that induce the germination of Orobanchaceae at concentrations as low as 10 pM [10–13]. Besides this, SLs act as recognition signals for symbiotic arbuscular mycorrhizal fungi and as plant hormones involved in a range of endogenous physiological processes, such as root architecture, leaf senescence, secondary growth, and response to environmental stresses [14–18]. The initial steps in SL biosynthesis are conserved across the plant kingdom and involve enzymes that convert β-carotene to carlactone (CL) [19]. *DWARF27* (*D27*) encodes a β-carotene isomerase that converts all-*trans*- to 9-*cis*-β-carotene. This is then converted into CL by CAROTENOID CLEAVAGE DEOXYGENASE 7 (CCD7) and CCD8, [20–23]. CL undergoes additional modifications to a range of different SLs [13, 24, 25]. The SLs orobanchol, orobanchyl acetate and fabacyl acetate have been previously reported in legume root exudates [11, 26].

In accordance with the role of SLs as Orobanchaceae germination stimulants, host genotypes displaying enhanced field resistance to Orobanchaceae, such as the rice cultivars NERICA, the faba bean breeding lines Navio and Quijote, and the pea breeding line ROR12, often secrete low SL levels in their root exudates [11, 27–29]. However, whether resistance of this germplasm is solely due to this low SL exudation has not been investigated. Indeed, it was suggested that the attachment of Orobanchaceae to their hosts can be prevented by SL-independent resistance mechanisms, involving other germination stimulants, germination inhibitors and host-derived haustorium initiation factors [30–35]. Additionally, post-attachment defense mechanisms to Orobanchaceae were documented [32, 36, 37]. The low-SL pea breeding line ROR12, displaying nearly complete resistance to *Oc* without an obvious yield penalty [11], is also likely to exhibit SL-independent resistance mechanisms, as it carries resistance alleles at three distinct quantitative trait loci (QTLs), previously named *PsOcr-1*, *PsOcr-2*, and *PsOcr-3* [38].

Loss-of function mutants of key SL biosynthetic genes have been reported in several species, including the pea mutant lines *rms1* and *rms5* [39, 40]. Although these mutants are expected to display enhanced resistance to Orobanchaceae, they exhibit severe pleiotropic phenotypes as a result of the complete loss of SLs, such as extreme branching and dwarfism, which makes them unsuitable for breeding purposes [22, 23, 41]. Notably, the response of SL biosynthetic mutants to Orobanchaceae was mostly assessed in vitro*,* by means of seed germination assays, rather than under field conditions [14, 23, 42, 43].

Here, we integrated liquid chromatography multiple reaction monitoring mass spectrometry (LC–MS/MS), RNA-seq and field trials to evaluate different pea germplasm, i.e. a global collection, the mutant lines *rms1* and *rms5*, and a set of recombinant inbred lines (RILs) originating from ROR12 as resistant parent. This allowed us to identify new sources of resistance to *Oc*, evaluate the relation between SL exudation and field resistance to *Oc*, and characterize QTLs underlying ROR12 resistance to *Oc.*

## Methods

### Field trials

Three pea lines, selected from the DISSPA-UNIBA germplasm collection, along with 133 lines from the U.S. Department of Agriculture (USDA) Pea Single Plant Plus (PSPP) collection (Supplementary Table 1), were evaluated over two growing seasons (2022–2023 and 2023–2024) (Supplementary Table 2 and Supplementary Table 3). The trials were conducted at the University of Bari (41°01′22.1″N 16°54′21.0″E), in a silty–clayey experimental field continuously cultivated with legumes and heavily infested by the *Oc* seed bank. Plants were sown according to randomized block designs with three blocks (2022–2023) or four blocks (2023–2024), which were placed orthogonally to a gradient of infestation observed in previous years. Each experimental unit consisted of two plants spaced 0.3 m apart along the row. Experimental units were spaced 1 m apart within the row and 1 m between adjacent rows. No fertilization and irrigation were applied during the crop cycle. Pest and pathogen management was carried out using single applications of deltamethrin, acetamiprid and difenoconazole, while weed control was performed with pendimethalin in pre-emergence and manual weeding in post-emergence. Response to *Oc* was evaluated by counting the number of parasitic shoots emerged aboveground per plant at crop maturity. To assess the homogeneity of *Oc* infestation, ten plants of the susceptible cultivar ‘Sprinter’ were randomly allocated in each block, and the standard deviation of the mean was calculated. Data on *Oc* emergence were normalized by square root transformation and further analysed by analysis of variance (ANOVA). As no significant genotype by year interaction was detected, data averaged over two years were used to obtain a frequency histogram of genotypic responses to *Oc*, using the ggplot2 and ggrepel R packages [44, 45].

Another field trial was conducted using the pea SL mutants *rms1* and *rms5* [39, 40]*,* provided by the French National Institute for Agriculture, Food, and Environment (INRAE) along with their wild-type genetic background (cv. Térèse), and the breeding line ROR12 [11]. Plants were sown in 2022 according to a randomized block design with seven blocks. Experimental units, consisting of five plants 0.2 m apart along the row, were spaced 1 m within and between rows. Crop management and *Oc* response evaluation were carried out as previously described. After data normalization by square root transformation and ANOVA, means were compared using the Tukey's Honest Significant Difference (HSD) post-hoc test implemented by the R package Agricolae, with significant level α set to 0.05 [46]. Finally, a box plot was obtained with the ggplot2 R package [44].

### Strigolactone quantification

Pea seeds were surface sterilized with 70% ethanol for 30 s and 2% sodium hypochlorite (v/v) for 30 min. They were then rinsed five times in sterile double-distilled water and kept in a petri dish on wet filer paper in the dark at 25 °C for two days to induce germination. Germinated seeds were transferred to pots filled with vermiculite and kept for one week. Afterwards, seedlings were moved to an aeroponic system arranged in a randomized block design with three replicates, in which each experimental unit consisted of a tank with eight plants. Plants were initially grown in 3 L of Hoagland’s nutrient solution with K_2_HPO_4_^.^3H_2_O (+ Pi) for 2 weeks, followed by K_2_HPO_4_^.^3H_2_O (–Pi) for another 10 days [47]. The aeroponic growing conditions were 23 °C during the day, 22 °C during the night, 16-h light/8-h dark photoperiod, and 60% relative humidity.

SL quantification was performed as previously described, with minor modifications [11]. Briefly, root exudates (1 L) were concentrated by loading onto a pre-equilibrated SPE C18-Fast column (500mg/3 mL, Grace Pure) and eluted with 3 mL 100% ethyl acetate. The solvent was evaporated in the nitrogen flow, and the dried extract was dissolved in 100 µl of acetonitrile:water (25:75, v:v) and filtered through a 0.22 μm filter for LC–MS/MS with MRM mode analysis. Chromatographic separation was performed using the Waters Acquity UPLC™ I-class system with Acquity UPLC™ bridged ethylene hybrid (BEH) C18 column (2.1 mm × 100 mm, 1.7 μm, Waters). This ultra-high pressure liquid chromatography (UHPLC) system is coupled to a Xevo® TQ-S quadrupole mass spectrometer with electrospray (ESI) ionization interface (Waters). The injection volume was set to 5 μl and analytes were eluted at a flow rate of 0.45 mL/min. Water + 15mM formic acid (A) and acetonitrile + 15mM formic acid (B) were used to generate a solvent gradient with the following profile: isocratic elution 0.4 min (15% B), 0.65 min (27% B), 5 min (40% B), 8 min (65% B), 9.5 min (95% B). The column was washed for one minute (95% B) and then equilibrated to initial conditions for 1.5 min (15% B). The ESI source was operated in positive mode and multiple reaction monitoring (MRM) was used to detect SLs using the following transitions: orobanchol_347 > 97, orobanchyl acetate_389 > 97, fabacyl acetate_405 > 97. Mass spectrometry data were processed using the MassLynx™ software (V4.2, Waters). Mass spectrometry data with a signal to noise ratio less than 10 were considered zero.

Statistical analyses for individual SLs were carried out using the Agricolae R package [46]. The Student’s t-test (α = 0.05) was used to compare the means of lines displaying contrasting response to *Oc*, or alternative alleles at the *PsOcr-1*, *PsOcr-2* and *PsOcr-3* QTLs. The ANOVA and the Tukey's HSD post-hoc test (α = 0.05) were performed to compare the lines *rms1*, *rms5* and Térèse. Data were graphically represented with barplots, which were obtained using the ggplot2 R package [44].

### RNA-seq analysis

ROR12, the *Oc* susceptible cultivar Sprinter and six F_6_ RILs originating from their cross were selected, based on available genotyping by sequencing (GBS) data and the KASP marker assays described by [38], to encompass all the 2^3^ possible homozygous configurations of resistance (R) and susceptibility (S) alleles at *PsOcr-1*, *PsOcr-2* and *PsOcr-3* (Table 1). Total RNA was extracted from root tissues using the TRI-Reagent with a Direct-zol RNA MiniPrep Kit, according to the manufacturer’s instruction (Zymo Research). RNA concentration, quality and integrity were checked using a NanoDrop 2000 UV–Vis spectrophotometer (Thermo Scientific) and standard gel electrophoresis. After total RNA isolation, complementary DNA (cDNA) libraries were prepared using the Hieff NGS Ultima Dual-mode mRNA Library Prep Kit for Illumina (Yeasen). In detail, mRNA was enriched using oligo(dT) beads, sheared using a fragmentation buffer and reverse transcribed into cDNA with random primers. After synthesizing the second strand, cDNA fragments underwent end-repair, poly(A) addition, and ligation to Illumina sequencing adapters. Size selection of the ligation products was performed using Hieff NGS DNA Selection Beads (Superior Ampure XP alternative|12601ES56, Yeasen). Ligated fragments were finally PCR amplified. Library sequencing was performed on an Illumina Novaseq 6000 platform (Illumina). Raw sequencing data were assessed for quality using FASTQC (https://www.bioinformatics.babraham.ac.uk/projects/fastqc) before and after trimming. The Trimmomatic software [48] was employed to remove sequencing adapters, low-quality bases (Phred < 25), short reads (< 35 nt), and the first 10 base pairs from each read. The kraken2/bracken pipeline [49] and a database comprising all bacteria and fungi species listed in RefSeq were used to assess potential contamination with pathogens. The STAR software [50] was used to index the *P. sativum* ZW6 reference genome [51] and to map the trimmed reads. Qualimap [52] was used to evaluate the mapping quality and infer strand specificity. Gene-level read summarization was conducted with FeatureCounts [53], focusing on reads with a mapping quality greater than 30 and counting fragments due to the paired-end nature of the data. The DESeq2 package [54] was used to normalize counts, filter out poorly expressed genes by setting a minimum of 10 counts in at least 3 samples, and to assess relations among samples by principal component analysis (PCA).

**Table 1:**
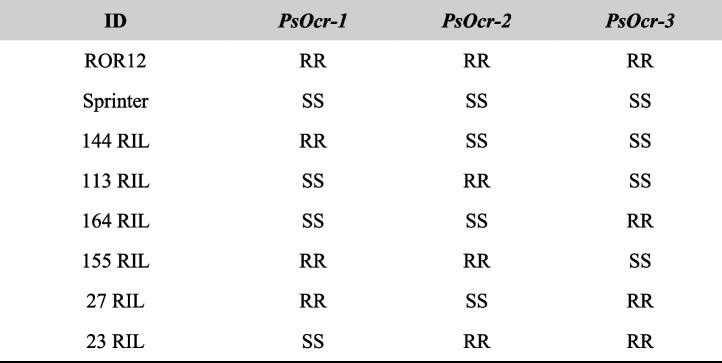
Homozygous genotypes at the QTLs *PsOcr-1*, *PsOcr-2* and *PsOcr-3* displayed by the breeding line ROR12, the cultivar Sprinter and six recombinant inbred lines (RILs) originating from their cross. S and R indicate the susceptibility and resistance allele, respectively

*RIL *Recombinant inbred line, *S *Susceptible allele, *R *Resistance allele

The DESeq2 package was also used to detect differentially expressed genes between lines carrying resistance (R) or susceptibility (S) alleles at each of the three previously identified QTLs *PsOcr-1*, *PsOcr-2* and *PsOcr-3* [38]. To reduce the number of statistically significant genes with a weak effect, the analysis was conducted using a testing log2 fold-change threshold of 0.5. After performing shrinkage of log2 foldchange (LFC) estimates with the *lfcShrink* function, genes with adjusted p-value < 0.01 and |log2FC|> 1 were considered differentially expressed genes (DEGs). DEGs within the *PsOcr-1*, *PsOcr-2* and *PsOcr-3* confidence intervals were identified by aligning the interval ends, previously determined on the Cameor genome [38, 55], on the newly published high-quality ZW6 genome [51]. Finally, a Volcano plot for each of the three comparisons was obtained using the EnhancedVolcano R package [56].

Enrichment analysis of DEGs for Gene Ontology–Biological Process (GO–BP) terms was performed using the hypergeometric test and the false discovery rate (FDR) multiple comparison correction implemented by the gProfiler2 R package (version 0.2.3) [57].

## Results

### Identification of new sources of resistance to *Oc* in pea

Preliminary screenings carried out on the pea ex situ germplasm collection held at the Department of Soil, Plant and Food Sciences, University of Bari (DISSPA-UNIBA), resulted in the detection of three lines, PS-00168, PS-00169 and PS-00300, showing low levels of *Oc* infection. These were further evaluated for two consecutive years in experimental fields severely infested by *Oc*, together with 133 lines from the Pea Single Plant Plus (PSPP) collection of the U.S. Department of Agriculture (USDA) (Supplementary Table 1). Within each experimental block, ten randomly allocated plants of the susceptible cultivar Sprinter displayed relatively low variation with respect to the average number of *Oc* shoots emerged per plant, indicating a fairly uniform level of *Oc* pressure within the blocks (Supplementary Table 4).

**Fig. 1:**
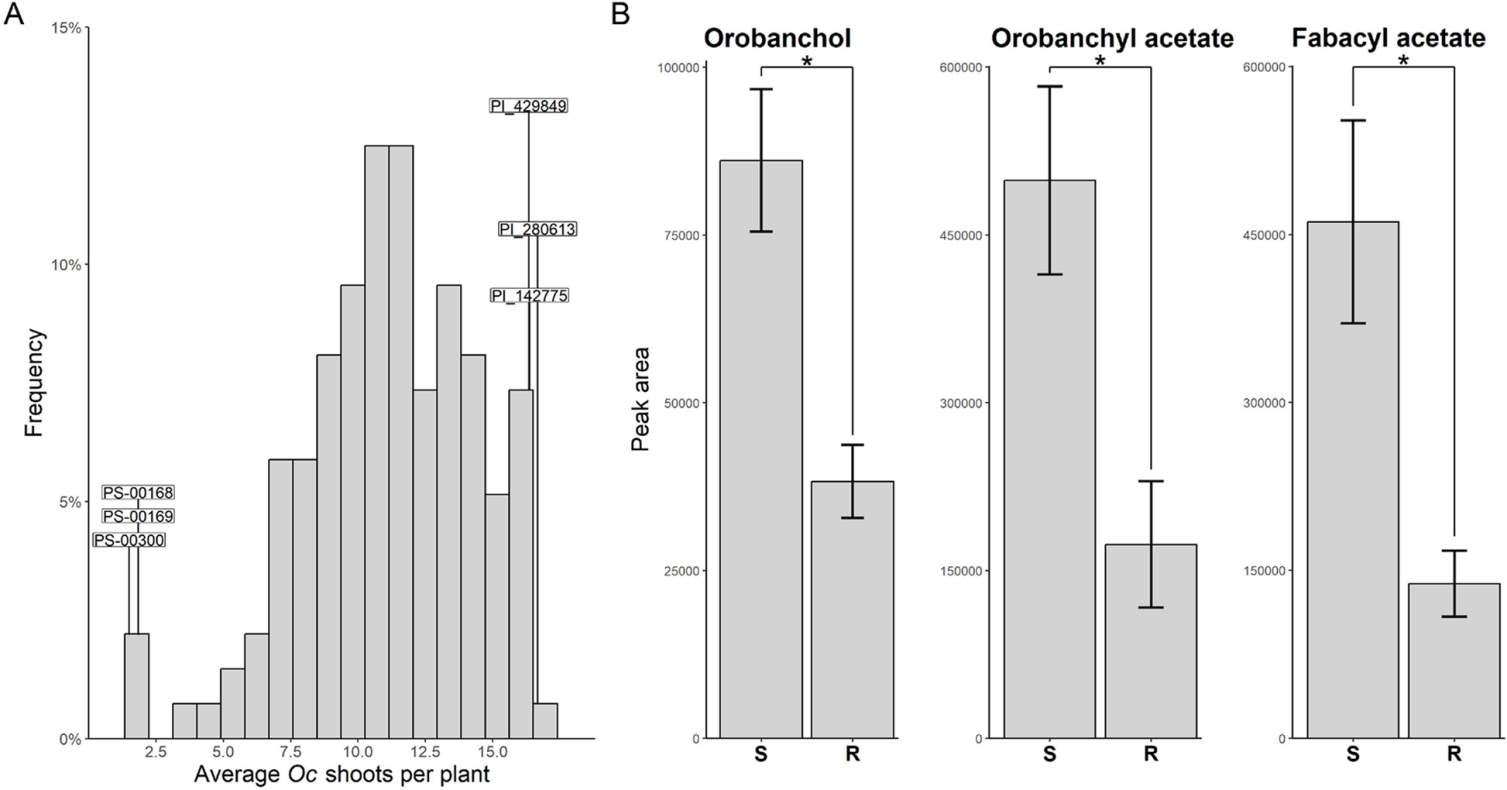
**A** Distribution of the number of O*robanche crenata* Forsk. (*Oc*) shoots emerged aboveground per host plant on a pea germplasm panel. Data are presented as the means of two growing seasons. Labels indicate six lines displaying extreme phenotypes, which were further analyzed for strigolactone quantification. **B** Orobanchol, orobanchyl acetate and fabacyl acetate peak areas associated with the root exudates of lines displaying resistant (R) and susceptible (S) phenotypes to *Oc*. Data are presented as means ± SE (*n* = 3). Asterisks indicate significant differences (*p* < 0.05, Student’s t-test)

The ANOVA indicated significant genetic variation for response to *Oc*, and non-significant genotype by year interaction (Supplementary Table 5). The lines PS-00168, PS-00169 and PS-00300 showed the highest level of resistance, with the average number of *Oc* shoots emerged per plant ranging from 1.5 (PS-00300) to 1.83 (PS-00168 and PS-00169) across the two years (Fig. 1A and Supplementary Table 6).

### Pea field resistance to *Oc* is associated with lower SL exudation

Aiming to study the relation between SL exudation and field resistance to *Oc*, we quantified SLs in root exudates collected from the resistant lines mentioned above and the lines PI_142775, PI_280613, and PI_429849. These showed the highest level of susceptibility, with the average number of *Oc* shoots emerged per plant ranging from 16.33 (PI_429849) to 16.67 (PI_280613) (Fig. 1A). Significantly lower levels of SLs were detected in the resistant group (Fig. 1B). Specifically, orobanchol, orobanchyl acetate and fabacyl acetate were reduced by 55.56%, 65.26% and 70.07%, respectively (Fig. 1B).

### Pea SL-deficient rms mutants only display partial resistance to *Oc*

**Fig. 2:**
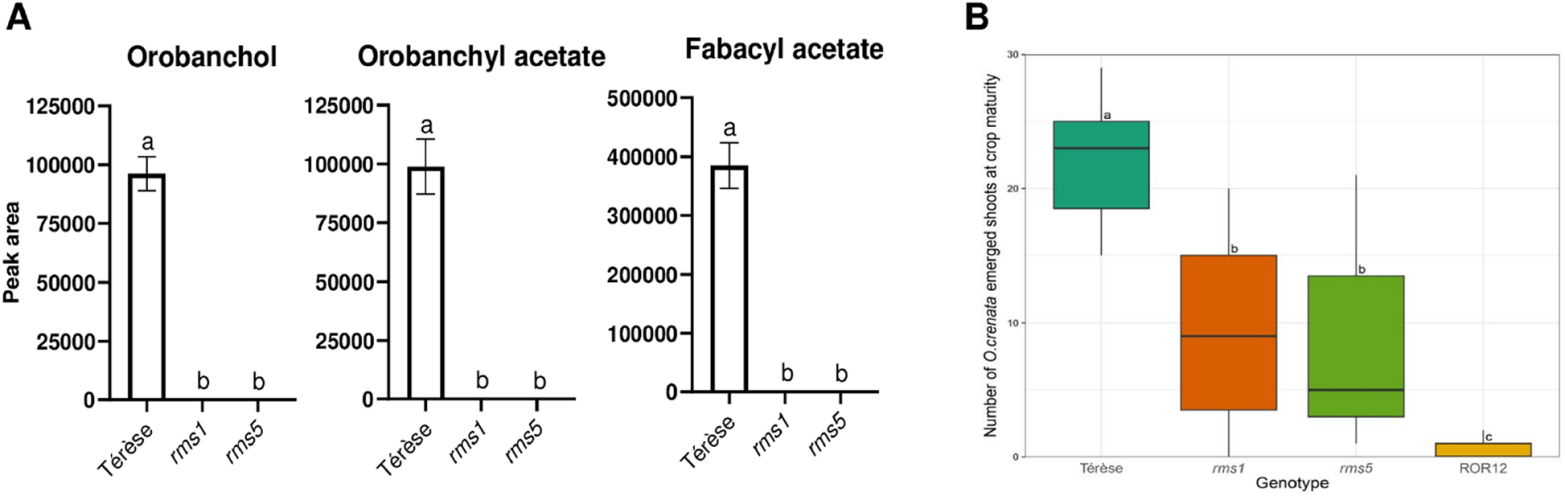
**A** Orobanchol, orobanchyl acetate and fabacyl acetate peak areas associated with the root exudates of the mutants *rms5* and *rms1*, and their genetic background (cv. Térèse). Data are shown as mean ± SE (*n* = 3). Different letters indicate significant differences (*p* < 0.05, Tukey’s HSD test). **B** Box plots showing the distribution of the number of O*robanche crenata* Forsk. (*Oc*) shoots emerged aboveground on Térèse, *rms1, rms5* and the breeding line ROR12. Data are presented as mean ± SE (*n* = 7). Different letters indicate significant differences (*p* < 0.05, Tukey’s HSD test)

To further explore the role of SLs in field resistance to *Oc*, we evaluated the previously reported *rms1* and *rms5* SL biosynthetic mutants [39, 40]. No SL was detected in the root exudates of these mutants, in contrast to their wild-type genetic background, i.e. the cv. Térèse (Fig. 2A). Surprisingly, both *rms1* and *rms5* displayed substantial parasitization, with an average of 9.43 and 8.57 *Oc* shoots emerged per plant, respectively, although they were significantly more resistant than Térèse, with an average of 22 *Oc* shoots emerged per plant (Fig. 2B). The level of *Oc* parasitization was not significantly different between *rms1* and *rms5* (Fig. 2B). Nearly complete field resistance was displayed by ROR12, associated with 0.5 *Oc* shoots emerged per plant on average (Fig. 2B).

### Both SL-dependent and SL-independent mechanisms play a role in the resistance to *Oc* displayed by the breeding line ROR12

The pea breeding line ROR12 carries *Oc* resistance alleles at three different QTLs, previously named *PsOcr-1* to *PsOcr-3* [38]. Based on available genotyping-by-sequencing data [38], we selected a panel of eight homozygous lines, i.e. ROR12, the *Oc* susceptible cultivar Sprinter and six F_6_ recombinant inbred lines derived from their cross, together encompassing the 2^3^ possible homozygous configurations of resistance (R) and susceptibility (S) alleles at the three QTLs (Table 1). These configurations were validated using Kompetitive Allele Specific PCR (KASP) marker assays previously developed on the QTL significance peaks [38] (Supplementary Fig. 1).

**Fig. 3:**
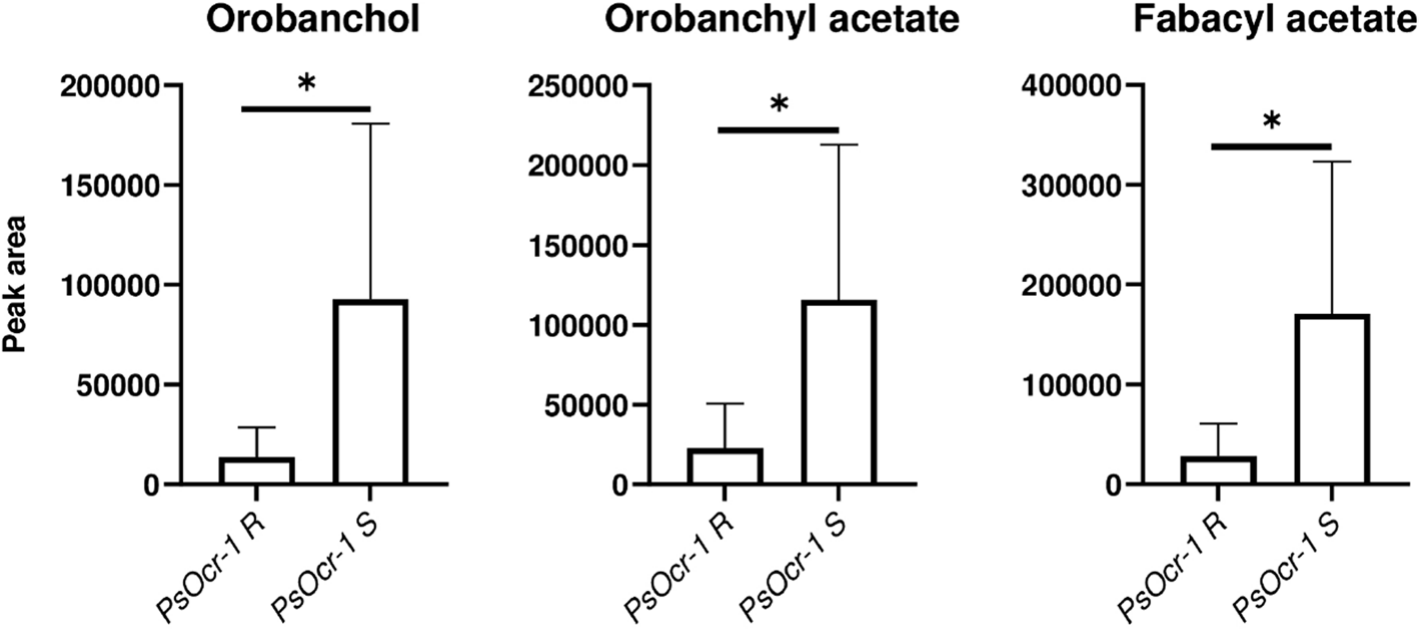
Orobanchol, orobanchyl acetate and fabacyl acetate peak areas associated with root exudates of RILs homozygous for the resistance (R) or the susceptibility (S) allele at *PsOcr-1*. Data are shown as means ± SE (*n* = 4). Asterisks indicate significant differences (*p* < 0.05, Student’s t-test)

The root exudates of lines carrying the *PsOcr-1* R allele displayed, compared to lines carrying the *PsOcr-1* S allele, significantly lower levels of orobanchol, orobanchyl acetate and fabacyl acetate, indicating that the resistance mechanism associated with *PsOcr-1* relates to lower SL exudation (Fig. 3). Notably, no significant difference in SL exudation was found by the analysis of lines carrying alternative alleles at either *PsOcr-2* or *PsOcr-3* (Supplementary Fig. 2), indicating that ROR12 resistance also involves SL-independent resistance mechanisms.

### Characterization of transcriptional profiles associated with different resistance mechanisms to *Oc*

To gain further insights into the molecular basis of ROR12 resistance to *Oc*, we performed RNA-seq to analyze gene expression in the roots of the eight pea lines above mentioned carrying different *PsOcr* allele combinations. Sequencing generated from 32.52 to 49.07 million raw reads per sample (Supplementary Table 7). Reads mapped onto the *P. sativum* ‘ZW6’ reference genome [51] ranged from 23.66 to 46.72 million, while uniquely mapped reads ranged from 67.41% to 92.26% (Supplementary Table 7). Principal component analysis showed a high level of consistency among the expression profile of biological replicates (Supplementary Fig. 3). Data from the cv. Sprinter were excluded from further analysis due to significant contamination with reads from *Fusarium* spp.

**Fig. 4:**
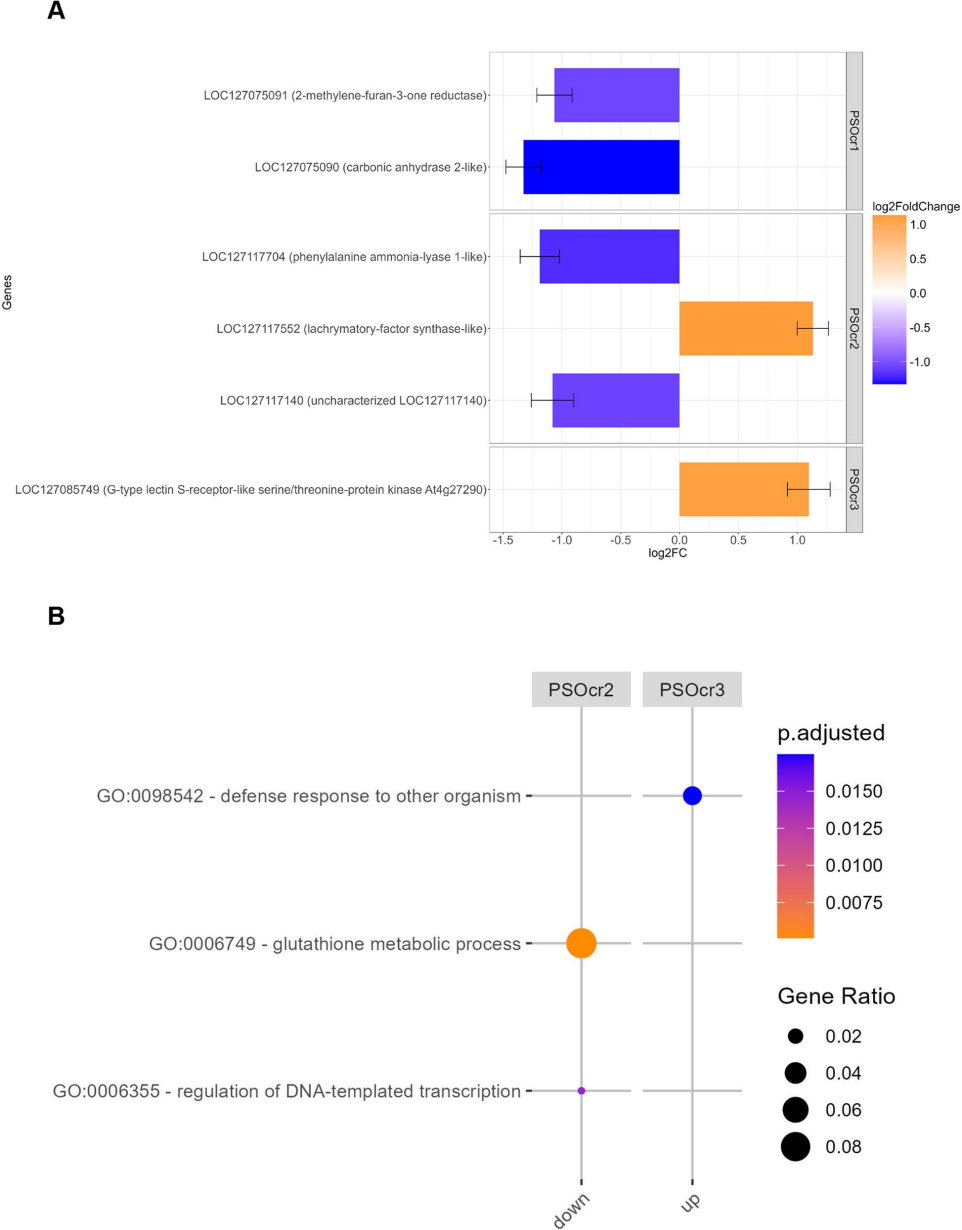
**A** Differentially expressed genes (DEGs) between lines homozygous for alternative alleles at the QTLs *PsOcr-1*, *PsOcr-2* and *PsOcr-3*. Bars indicate DEGs within QTL confidence intervals. The bar colors represent log_2_ fold changes (LFC) value, according to a blue (down-regulated)/orange (up-regulated) scale. **B** Gene ontology enrichment analysis for DEGs between lines homozygous for alternative alleles at *PsOcr-2* and *PsOcr-3*. Dot sizes indicate the proportion of DEGs in each set, while dot colors indicate the significance level, according to a blue (less significant)/orange (more significant) scale

Comparing lines homozygous for alternative alleles at *PsOcr-1* led to the identification of 15 differentially expressed genes (DEGs) (Supplementary Fig. 4A and Supplementary Table 8). Two of them, encoding a 2-methylene-furan-3-one reductase and a carbonic anhydrase 2-like protein, resided in the *PsOcr-1* confidence interval and were downregulated in lines carrying the R allele (Fig. 4A). As for lines carrying alternative alleles at *PsOcr-2,* 108 DEGs were found (Supplementary Fig. 4B and Supplementary Table 8). Among them, three genes resided in the *PsOcr-2* confidence interval, including two (encoding a phenylalanine ammonia-lyase 1-like protein and an uncharacterized proteins) down-regulated and one (encoding a lachrymatory-factor synthase-like protein) up-regulated in lines carrying the R allele (Fig. 4A). Finally, 54 DEGs were found between lines carrying alternative alleles at *PsOcr-3* (Supplementary Fig. 4C and Supplementary Table 8). One of them, encoding a G-type lectin S-receptor-like serine/threonine-protein kinase, resided in the *PsOcr-3* confidence interval and was up-regulated in lines carrying the R allele (Fig. 4A).

DEGs between lines differing for the allelic configuration at *PsOcr-1* were not significantly enriched for any gene ontology (GO) biological process (BP) term. In contrast, the GO BP terms GO:0006749 and GO:0006355, indicating glutathione metabolic process and regulation of DNA-templated transcription, respectively, were significantly enriched in DEGs between lines carrying alternative allelic configurations at *PsOcr-2* (Fig. 4B). Finally, the GO BP term GO:0098542, indicating defense response to other organisms, was enriched in DEGs between lines carrying alternative allelic configurations at *PsOcr-3* (Fig. 4B).

## Discussion

We report the identification of three pea lines, PS-00168, PS-00169, and PS-00300, representing novel sources of resistance to *Oc* (Fig. 1A), a root parasitic weed severely affecting pea cultivation in the Mediterranean region. Notably, all the lines mentioned above, as well as the previously described *Oc*-resistant line ROR12 [11], originate from southern Italy (Supplementary Table 1). This geographic clustering raises important questions regarding the origin, spread, and potentially shared genetic basis of resistance within this regional germplasm.

The characterization of pea genotypes with contrasting response to *Oc* showed that pea field resistance is commonly associated with low SL exudation (Fig. 1B), in line with previous studies relating low SL exudation with resistance to Orobanchaceae in rice and faba bean [27, 28]. Another SL-dependent mechanism of resistance to Orobanchaceae, reported in sorghum and maize, involves qualitative differences in SL composition, as individual SLs may differ in their activity as germination stimulants [12, 58, 59]. However, this mechanism does not appear to apply to PS-00168, PS-00169, PS-00300, which show similar ratios among the three pea SLs orobanchol, orobanchyl acetate and fabacyl acetate compared to susceptible germplasm (Fig. 1B).

Although low SL exudation is known to reduce germination of Orobanchaceae [13], its translation into effective field resistance has been poorly explored. Indeed, SL-biosynthetic mutants have been primarily tested by in vitro germination assays or by pot assays [42, 43, 60, 61]. The only exceptions are represented by the tomato mutants *sb1* and *sb2*, which carry loss-of-function mutations in the *CCD7* and *CCD8* genes, respectively, and were recently reported to exhibit complete field resistance to the broomrape species *Phelipanche aegyptiaca* [62, 63]. In contrast, as a key novelty of this study, we show that compared with their wild-type genetic background, the SL biosynthetic mutants *rms1* and *rms5*, which do not produce detectable SL levels, only show a moderate reduction in *Oc* parasitization under field conditions (Fig. 2B). This highlights the limitations of relying solely on SL exudation as a breeding strategy to introduce *Oc* resistance in pea. Our findings are in line with earlier in vitro observations, showing significant germination of *Oc* on *rms1* root exudates [23]. Incomplete resistance of *rms* mutants could be attributed to the presence of non-SL germination stimulants, which have been previously reported in pea root exudates [30, 35].

Consistent with the partial contribution of SL deficiency to resistance, we showed that only one of the three QTLs associated with the nearly complete field resistance of ROR12, *PsOcr-1,* is correlated with lower SL exudation (Fig. 3 and Supplementary Fig. 2). Nevertheless, no known SL biosynthetic or catabolic gene is located within the *PsOcr-1* confidence interval, nor is differentially expressed between lines with alternative alleles at *PsOcr-1*. However, one of the two DEGs in *PsOcr-1* encodes a 2-methylene-furan-3-one reductase. Although there are no reports on a possible role of 2-methylene-furan-3-one reductases in the modification of SLs, the strigolactone D-ring is a furanone. In addition, non-SL furanones, termed debranones, mimic SLs in inhibiting shoot branching and stimulating the germination of *Striga* and *Orobanche* [64–66]. Functional analysis of this gene could clarify its potential impact on SL activity or mimicry.

Within *PsOcr-2*, a differentially expressed gene encodes a phenylalanine ammonia-lyase (PAL), a well-known enzyme associated with response to various biotic and abiotic stresses [67, 68]. *PAL* expression is induced upon infection by the root parasitic plant species *Striga hermonthica*, *Orobanche foetida* and *Oc* in rice, chickpea and faba bean [69–71], respectively, making it a strong candidate gene underlying *Oc* resistance in pea.

Finally, the sole DEG within the *PsOcr-3* confidence interval encodes a G-type lectin S-receptor-like serine/threonine-protein kinase*.* Increasing evidence supports the role of lectin receptor-like kinases (LecRLKs) in the activation of defense pathways against pathogens [72]. In addition, the RLK HAOR7 in sunflowers provides resistance against *O. cumana* [73]. Furthermore, lines carrying contrasting alleles at *PsOcr-3* show significant enrichment of the GO-BP term *defense response to other organism*, suggesting that this QTL may contribute to enhanced defense signaling.

Taken together, our results provide evidence that achieving substantial field resistance to *Oc* in pea requires the integration of low SL exudation with SL-independent resistance mechanisms. It will be particularly interesting to investigate whether this finding also applies to other legumes affected by *Oc* or to crops targeted by other Orobanchaceae species. The identification of candidate genes within resistance QTLs offers promising avenues for downstream functional studies. To this end, we are currently employing TILLING to functionally characterize the role of selected candidates. Additional approaches, such as conventional mutagenesis and recently established genome editing technologies in pea [74, 75], are also feasible and may complement our efforts.

## Conclusions

We identified three new potential sources of field resistance to *Oc* in pea. This finding is highly relevant, as no commercially available cultivars currently provide resistance to this parasitic species, which remains a major constraint on pea cultivation in Mediterranean agro-ecosystems.

By integrating SL quantification with field phenotyping of diverse genetic resources, we demonstrated that low or absent SL exudation can confer only partial resistance to *Oc*. This highlights the need to expand breeding strategies beyond SL-based mechanisms, thus targeting additional SL-independent pathways.

Our results not only provide valuable information for pea improvement, but also offer new insights into the genetic complexity of resistance to parasitic Orobanchaceae. The identification of DEGs within QTLs associated with resistance points to molecular mechanisms potentially involved in both SL-dependent and independent responses. These findings lay the groundwork for functional validation of these genes by reverse genetics approaches.

Finally, the conceptual framework and methodologies applied in this study may be extended to other legume species and crops affected by Orobanchaceae, thereby broadening the impact of our research in the context of sustainable agriculture and parasitic weed management.

## Supplementary Information

Supplementary Material 1. Supplementary Figure 1. KASP assays for the *PsOcr-1* (A), *PsOcr-2* (B), and *PsOcr-3* (C) quantitative trait loci (QTLs) associated with pea resistance to *O. crenata*. Supplementary Figure 2. Orobanchol, orobanchyl acetate and fabacyl acetate peak areas associated with the root exudates of RILs homozygous for the resistance (R) or susceptibility (S) allele at (A) *PsOcr-2 *and (B) *PsOcr-3.* Data are shown as means ± SE (*n *= 4). ns indicate no significant difference (p >0.05, Student’s t-test). Supplementary Figure 3. Principal component analysis for the transcriptional profiles of ROR12, Sprinter and six recombinant inbred lines originating from their cross. Dots of the same color indicate biological replicates of the same genotype (*n *= 3). Supplementary Figure 4. Volcano plots showing differentially expressed genes between lines homozygous for the resistance (R) and susceptibility (S) allele at (A) *PsOcr-1*, (B) *PsOcr-2*, and (C) *PsOcr-3.*

Supplementary Material 2. Supplementary Table 1. Information on the germplasm panel evaluated in this study for response to *O. crenata*. Supplementary Table 2. Climatic data for the field trial conducted in 2022–2023. Supplementary Table 3. Climatic data for the field trial conducted in 2023–2024. Supplementary Table 4. Number of *O. crenata* shoots emerged on the cultivar Sprinter within each experimental block of the field trials carried out in 2022-2023 and 2023-2024. Data are presented as means ± SD. Supplementary Table 5. ANOVA for response to *O. crenata* of the pea germplasm panel evaluated in this study. Supplementary Table 6. Mean infestation data of *O. crenata* recorded on the pea germplasm panel over two growing seasons (2022–2023 and 2023–2024). Means were calculated using three replicates in 2022–2023 and four replicates in 2023–2024. Supplementary Table 7. Summary for the results of the RNA-seq experiment Supplementary Table 8. List of differentially expressed genes between lines carrying resistance (R) and susceptibility (S) allele at *PsOcr-1*,*PsOcr-2*, and *PsOcr-3*.

## Data Availability

The datasets used and analyzed during the current study are available in the supplementary information files and from the corresponding authors on reasonable request.
