# Supplementary material for: Field resistance to Orobanche crenata in pea (Pisum sativum L.): beyond strigolactones
Source: BMC Plant Biol. 2025 Oct 8;25:1340. doi: 10.1186/s12870-025-07296-x (PMC12505547; doi:10.1186/s12870-025-07296-x)
Supplement: Supplementary file 1 — Supplementary Material 1. Supplementary Figure 1. KASP assays for the PsOcr-1 (A), PsOcr-2 (B), and PsOcr-3 (C) quantitative trait loci (QTLs) associated with pea resistance to O. crenata. Supplementary Figure 2. Orobanchol, orobanchyl acetate and fabacyl acetate peak areas associated with the root exudates of RILs homozygous for the resistance (R) or susceptibility (S) allele at (A) PsOcr-2 and (B) PsOcr-3. Data are shown as means ± SE (n = 4). ns indicate no significant difference (p >0.05, Student’s t-test). Supplementary Figure 3. Principal component analysis for the transcriptional profiles of ROR12, Sprinter and six recombinant inbred lines originating from their cross. Dots of the same color indicate biological replicates of the same genotype (n = 3). Supplementary Figure 4. Volcano plots showing differentially expressed genes between lines homozygous for the resistance (R) and susceptibility (S) allele at (A) PsOcr-1, (B) PsOcr-2, and (C) PsOcr-3. [file 12870_2025_7296_MOESM1_ESM.pdf]

Supplementary Figure 1

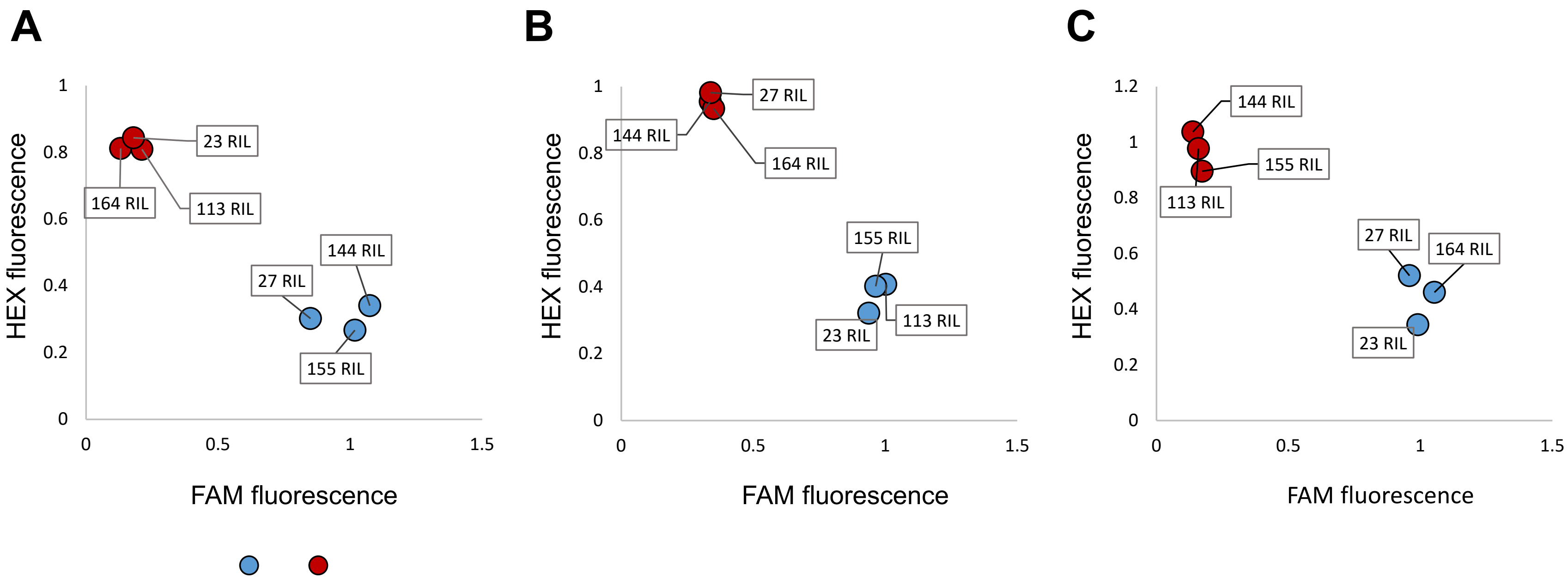

Supplementary Figure 1. KASP assays for the *PsOcr-1* **(A)**, *PsOcr-2* **(B)**, and *PsOcr-3* **(C)** quantitative trait loci (QTLs) associated with pea resistance to *O. crenata*.

Supplementary Figure 2

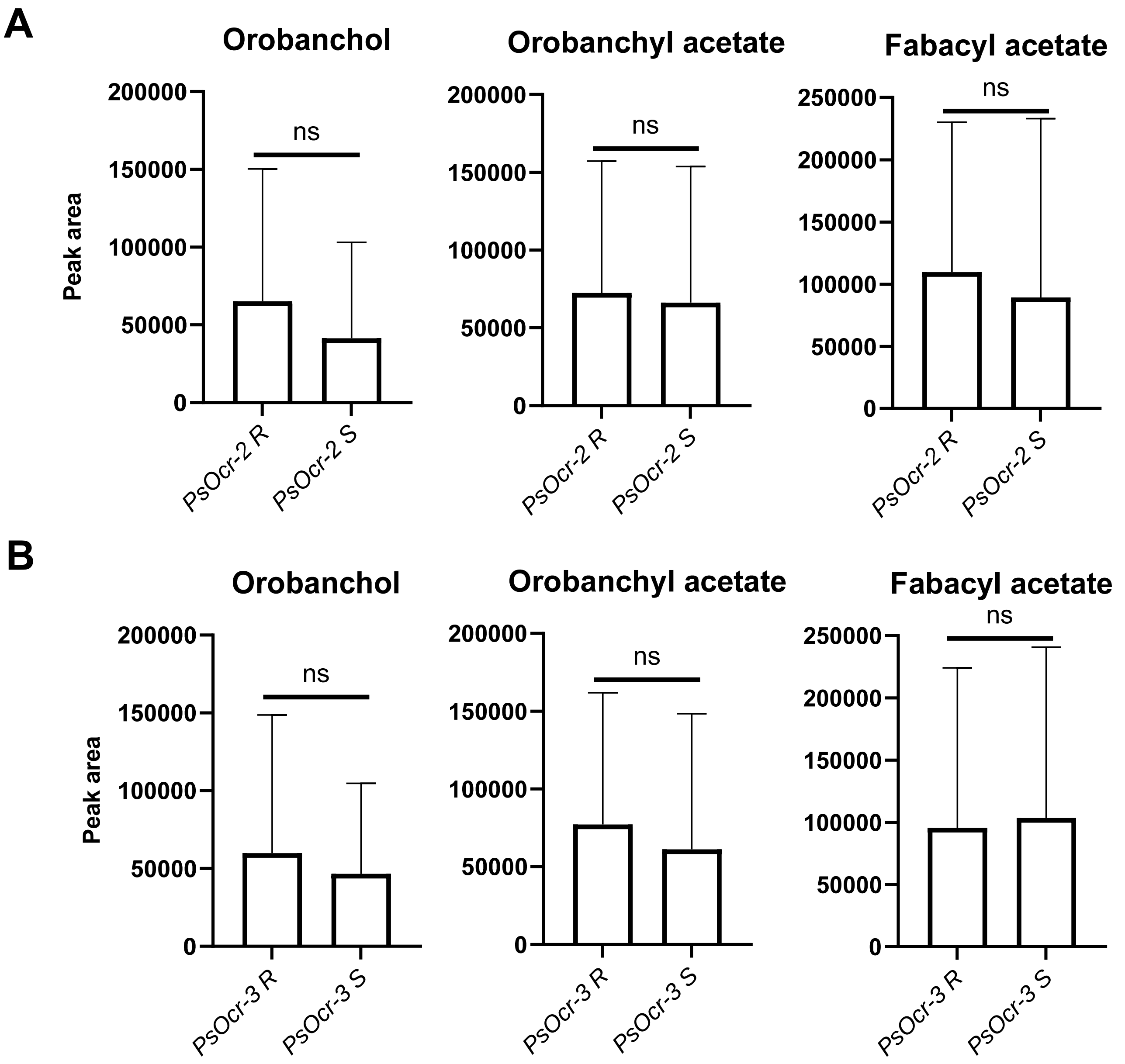

Supplementary Figure 2. Orobanchol, orobanchyl acetate and fabacyl acetate peak areas associated with the root exudates of RILs homozygous for the resistance (R) or susceptibility (S) allele at **(A)** *PsOcr-2* and **(B)** *PsOcr-3.* Data are shown as means ± SE (n = 4). ns indicate no significant difference ($p > 0.05$, Student's t-test)

Supplementary Figure 3

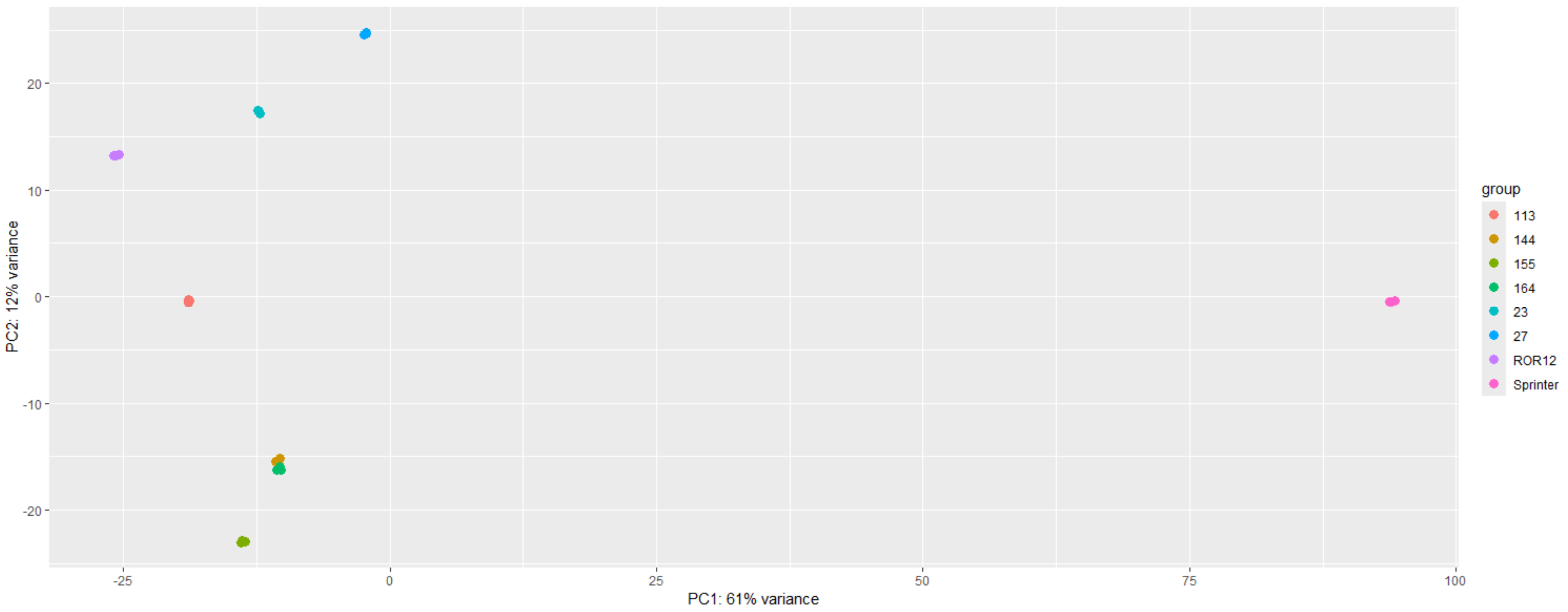

Supplementary Figure 3. Principal component analysis for the transcriptional profiles of ROR12, Sprinter and six recombinant inbred lines originating from their cross. Dots of the same color indicate biological replicates of the same genotype (*n* = 3).

Supplementary Figure 4

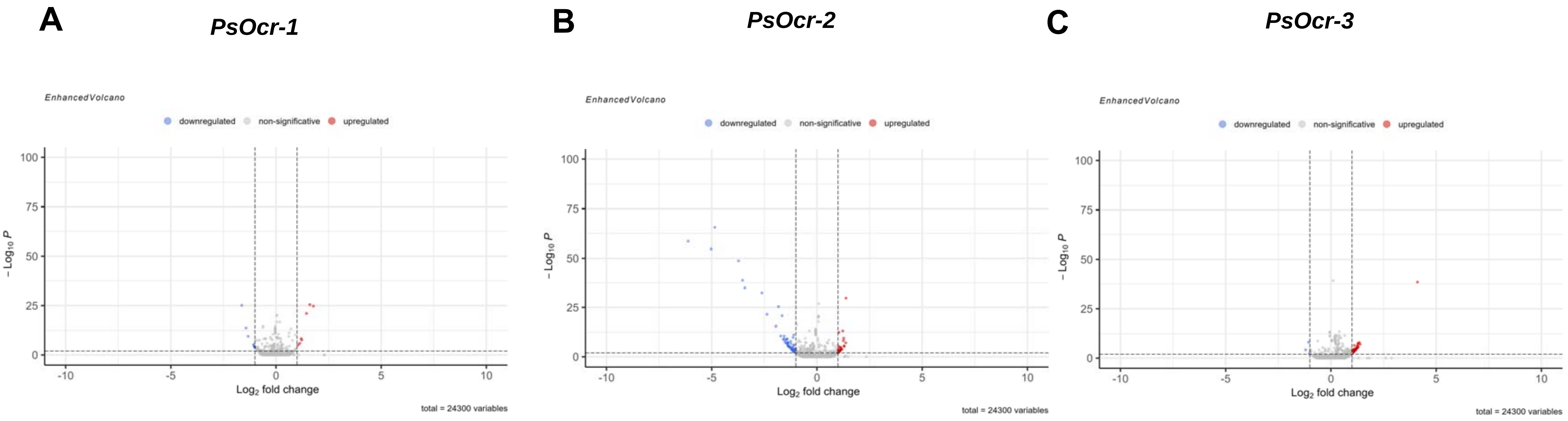

Supplementary Figure 4. Volcano plots showing differentially expressed genes between lines homozygous for the resistance (R) and susceptibility (S) allele at **(A)** *PsOcr-1*, **(B)** *PsOcr-2*, and **(C)** *PsOcr-3*.
